# A simple questionnaire to detect chronic kidney disease patients from Long An province screening data in Vietnam

**DOI:** 10.1186/s13104-017-2847-7

**Published:** 2017-10-30

**Authors:** Huong T. B. Tran, Thu T. N. Du, Nhat D. Phung, Ninh H. Le, Toan B. Nguyen, Hai T. Phan, De T. Vo, Edgar L. Milford, Sinh N. Tran

**Affiliations:** 1 Nephrology Division, Department of Medicine, University of Medicine and Pharmacy, 217 Hong Bang Str., Dist 5, Ho Chi Minh City, Vietnam; 2 Urology Department, Cho Ray Hospital, Ho Chi Minh City, Vietnam; 3 Institute of Public Health, Ho Chi Minh City, Vietnam; 4 MEDIC Medical Center, Ho Chi Minh City, Vietnam; 5 Health Department, Long An Province Tan An City, Vietnam; 6 Renal Division, Dept. Medicine, Brigham and Women’s Hospital, Harvard Medical School, Boston, MA USA; 7 Urology Department, University of Medicine and Pharmacy, Ho Chi Minh City, Vietnam

**Keywords:** Chronic kidney disease, Albuminuria, eGFR, Risk score, Survey, Screening, Prevalence

## Abstract

**Background:**

The prevalence of chronic kidney disease (CKD) in rural Vietnam is unknown. We wished to determine the prevalence of CKD and determine whether a simple questionnaire was able to detect individuals at high risk of CKD before expensive confirmatory laboratory testing.

**Methods:**

A cross sectional study was performed. We recruited 2037 participants from 13 communes of Long An province, Vietnam, for CKD screening with urine albumin/creatinine ratio (ACR) measured by immunoturbidimetric method and serum creatinine to estimate glomerular filtration rate (eGFR). CKD was defined as either ACR ≥ 30 mg/g or eGFR _MDRD_ < 60 ml/min/1.73 m^2^. A two page questionnaire with 23 variables was administered to each participant with queries postulated to be correlated with risk of CKD.

**Results:**

Of the 2037 participants, 260 (12.76%) were found to have CKD. Five questionnaire variables (age more than 50, measured hypertension, history of diabetes, history of hypertension, and history of a low salt diet) were correlated with CKD, and used to construct a risk score for CKD.

**Conclusions:**

CKD is common in Vietnam. Our questionnaire and risk score tool can be used to detect individuals with a higher likelihood of CKD, and who can then be more economically screened with routine laboratory confirmatory tests.

## Background

Chronic kidney disease (CKD) is a condition associated with substantial morbidity, mortality and economic loss [1–3]. The prevalence of CKD in different studies varies by the definition, measure used, study design, and study population [2, 3]. Kidney Early Evaluation Programs (KEEP) surveys in the United States, Japan, and Mexico reported prevalence of CKD of 27.1, 26.7, and 22% respectively, defined as impaired estimated glomerular filtration rate (eGFR), or proteinuria among high risk patients (history of hypertension, diabetes, family history of hypertension, diabetes and kidney disease) [4–7]. In the general populations, the prevalence of impaired kidney function (eGFR less than 60 ml/min/1,73) varied between 1.7 and 5.2% [2]. In a survey of CKD in Hatay, Vietnam, 3.6% of subjects more than 40 years old had eGFR less than 60 ml/min/1.73 m^2^ (estimated by the Modification of Diet in Renal Disease (MDRD) formula, adjusted by the Japanese racial coefficient) [8]. Geographic, racial, and economic factors influence the prevalence of CKD [9, 10]. In Vietnam, non-communicable diseases such as stroke, cardiovascular diseases, cancer, and diabetes, have risen to 60% of disease related morbidity yearly [11]. In 2010, approximately 60 million Vietnamese (71.2% of the general population and 90% of the poor) live in rural areas [12]. Access to routine laboratory screening of healthy people for CKD is not economically feasible in rural areas of Vietnam. Untargeted screening of the rural populations is not cost-effective. Prior probability of disease must be high in order to justify the resources needed for confirmatory laboratory testing. An inexpensive paper survey instrument, the results of which can identify individuals at high risk of disease, is desirable. The Screening for Occult REnal Disease (SCORED) study from the United States [13] was an example of an approach in which nine variables were predictive of CKD. Another study from Thailand used 4 variables (age, diabetes, hypertension and kidney stone) to predict CKD [14]. We now report on a study (Screening for Training and Early Detection and Prevention of Chronic Kidney Disease, STEP study) in Long An province, a rural area of Vietnam, to assess the prevalence of CKD and to develop a model for detection of persons at high risk of CKD on the basis of a simple questionnaire and blood pressure measurement.

## Methods

### Study area

Long An province, located in the Southwest of Vietnam, about 60 km from Ho Chi Minh City, has a population of 1,477,300 in 2014 with an average population density of 329/km^2^ [15]. Long An is situated in a low lying coastal region and serves as a bridge between Ho Chi Minh City in the North and 12 provinces of the Mekong Delta in the South. At the time of the study, the public health system in Long An consisted of 3 provincial hospitals, one multidisciplinary hospital, one tuberculosis hospital, and a traditional medicine hospital [15]. Secondary elements of this health care network included 13 district hospitals, 5 regional polyclinics and 192 commune health stations. The commune health station is the basic unit of health care delivery for rural areas of Vietnam.

### Sampling design

**Fig. 1:**
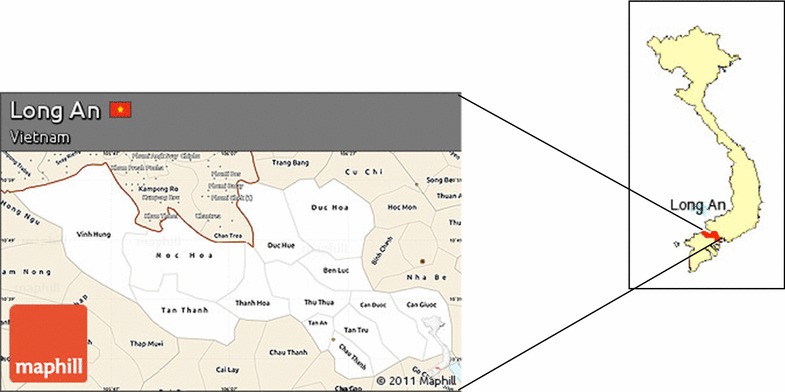
Long An map. We state that the source of the Long An map we used in Fig. 1 is from the website for free map of Maphill: http://www.maphill.com/vietnam/long-an/simple-maps/political-map/single-color-outside/free/

This cross sectional study was conducted in Long An province, Vietnam. Long An was divided into 14 “communes” (13 districts and 1 capital city survey unit, Tan An), using the National Population Census of 2009 as a guide. Eight of the communes (Tan An, Thu Thua, Ben Luc, Duc Hoa, Chau Thanh, Tan Tru, Can Duoc, and Can Giuoc) were used as regions for sampling. The remaining 6 communes with sparsely population at flood area (Tan Hung, Vinh Hung, Moc Hoa, Tan Thanh, Thanh Hoa, and Duc Hue) were combined into a single region for sampling (Fig. 1). Prior to conducting the study, we estimated the prevalence of CKD to be 11.3% with a marginal error of 2% (5% probability of type I error) and estimated number needed to screen of 2000. We used the “proportional to population size” method to obtain samples that were representative of the populations of the 9 regions, and used 14 villages/towns (called “kidney camps”) to recruit. The study group obtained resident lists for the target villages. 3053 invitation letters were distributed to residents based on gender and age demographics of the province. Subjects younger than 19, pregnant women and those menstruating at time of sample collection were excluded. The recruitment was discontinued when we reached the number needed to screen. 2062 adults from 13 villages/towns ultimately participated in our survey, and 2037 individuals completed the study. Each kidney camp included 2–3 nephrologists, 2–3 urologists, 4–5 residents, 3–4 nurses, 1–2 epidemiologists, and 8–10 medical students. This study was approved by the ethics committee of the Ho Chi Minh City Urology and Nephrology Association, the Institute of Public Health Ho Chi Minh City, The MEDIC Medical Center and Health Department of Long An province. Each participant also gave verbal informed consent for their participation.

### Questionnaire and data collection

The two page questionnaire with 25 questions included categorical variables as self-reported education level (from illiterate to highest education level), place of birth (at home, local health care or hospital), past and current tobacco use, alcohol use (more than one glass three times weekly), low salt diet (avoid adding salty condiments and salt on table), exercise (two or more times per week regularly). Other dichotomous variables as marital status, personal and family histories of diseases diagnosed by previous physician as kidney disease, kidney stone, urinary tract infection, diabetes, hypertension, stroke, cardiovascular disease, congestive heart failure, and amputation secondary to medical disease. All participants were personally interviewed by physicians using standardized language, followed by physical examination. Any abnormal clinical findings requiring treatment and follow-up were referred to the commune health stations, or Long An Hospital. Clinical examination included weight, height, waist to hip circumference ratio, and blood pressure measurement. Blood pressure was obtained in sitting position after a 10 min rest.

### Blood and urine sample collections

Morning blood and urine samples were collected and analyzed on the same day at the MEDIC Medical Center at Ho Chi Minh City. Serum and urine creatinine were measured by Kinetic Alkaline Picrate technique and urine albumin measured by immunoturbidimetric method (Abbott Architect CI systems). Spot urine albumin/creatinine ratios (ACR) were calculated and ACR ≥ 30 mg/g was used as the threshold for CKD recommended by the American Diabetes Association 2012 [16] and the Kidney Disease Improving Global Outcome 2012 [17]. The participants with abnormal lab tests on the first screening were asked to repeat those tests within 3 months.

### Definition of variables

Chronic kidney disease was defined as either ACR ≥ 30 mg/g or eGFR _MDRD_ < 60 ml/min/1.73 m^2^. The eGFR was calculated using the 4 variable MDRD equation. eGFR _MDRD_ (ml/min/1.73 m^2^) = 175 × (S_cr_)^−1.154^ × (Age)^−0.203^ × (0.742 if female). There were no subjects of African descent in the survey. Hypertension at screening was defined as mean systolic blood pressure ≥ 140 mmHg, or diastolic blood pressure ≥ 90 mmHg on three measurements. Increased waist circumference was defined by waist circumference > 90 cm (men) or > 80 cm (women), per the International Diabetes Federation (IDF) cut off points for South Asians, Chinese and Japanese [18]. Nocturia was defined as urination more than two times per night, hematuria as patient report of visible blood in the urine, anemia as a history of prior blood transfusion.

### Statistical analysis

Independent variables from the questionnaire as well as demographic information and laboratory results were entered into a table for statistical analysis. All analyses were performed using JMP-Pro^®^ statistical software, version 11.0 (SAS Institute, Cary, NC). Logistic regression was used to create the prediction model with CKD as a binary outcome. We first analyzed the univariate associations between the independent variables and CKD. We used stepwise logistic regression and backward elimination to reach the final model in which all the predictors in the model were significant at p < 0.001 and with an odds ratio of > 1.7. Using the regression coefficients, we estimated the patient-specific probability of having CKD. The probability of CKD was calculated by the formula 1/(1 + e^−A^), in which A depended on the final statistic significant variables and their responsible β coefficients (A = β0 + β1 × 1 + β2 × 2· · · + βn × n). A probability for confirmed laboratory CKD was chosen to make the discreet patterns of “yes” and “no” from significant questionnaire variables. The utility of the prediction model was evaluated based on several measures: percentage of positive cases, sensitivity, specificity, positive predictive value, and area under the receiver operator characteristic (ROC) curve. The maximum of area under the curve (AUC) = 1, meant the diagnostic test was perfect to differentiate between the diseased and non-diseased subjects. While AUC = 0.5, meant this differentiation was by chance. The number of persons needed to do lab tests to find 1 CKD subject = 1/(percentage of CKD within each group/100). The cost effectiveness per CKD case with or without using questionnaire before confirmatory laboratory tests was calculated.

## Results

### Prevalence of chronic kidney disease

Table 1 shows the demographics of the sample used in this study. The distribution of age groups of this sample was not statistically different from the distribution in Long An population in the latest Vietnamese National Census of 2009 (Pearson Chi square = 2.2, p = 0.3). However, the proportion of females in this sample was higher than in the Census (54.39% vs. 51.59%) (Pearson Chi square = 6.4, p = 0.01). 68.44% of the participants were less than 50 years old. Of 577 individuals with hypertension on screening, 239 (41.4%) subjects had history of hypertension. Two-hundred-sixty subjects in our study (260/2037, 12.76%) had CKD, 232/2037 (11.4%) had ACR ≥ 30 mg/g, and 48/2037 (2.4%) had an eGFR < 60 ml/min/1.73 m^2^ (Table 2). We examined the correlation between stage of CKD, determined by eGFR, and the degree of albuminuria (Table 2). We found a correlation between stage of CKD and albuminuria. (Pearson Chi Square 166, p < 0.0001; Fisher’s Exact Test p < 0.0001). All of the subjects with both low GFR and albuminuria were 40 years old or more. Indeed, 47/48 patients with low eGFR were ≥ 40 years old. While 8–12% of the subjects with stage 1 or 2 CKD had albuminuria, a full 41.7% of individuals with stage 3–5 CKD had albuminuria. Stage 3–5 CKD was found in 8.6% of 232 individuals with albuminuria compared to 1.6% of 1805 individuals without albuminuria (Table 3).

**Table 1:** Demographic data

Variable	N	Mean ± SD or %
Age (years)	2037	42.3 ± 14.2
19 to 29	423	20.8%
30 to 39	604	29.6%
40 to 49	367	18.0%
50 to 59	385	18.9%
60 to 69	167	8.2%
More than 70	91	4.5%
Men (n, %)	929	45.6%
Height (cm)	2037	158.5 ± 8.2
Weight (kg)	2037	54.9 ± 9.6
Body mass index (kg/m^2^)	2037	21.5 ± 3.3
Waist circumference (cm)	2037	74.1 ± 9.1
Hip circumference (cm)	2037	88.8 ± 7.0
Waist hip ratio	2037	0.82 ± 0.1
Increased waist circumference (n, %)	323	15.8%
Hypertension on screening (n, %)	577	28.3%
Mean blood pressure (mmHg)	2037	95.4 ± 13.5
Serum creatinine (mg/dL)	2037	0.89 ± 0.29
eGFR _MDRD_ (ml/min/1.73 m^2^)	2037	87.7 ± 15.8
Albumin/creatinine ratio (mg/g)*	2037	8.89 [4.75, 15.38]

Body mass index = Weight (Kg)/(Height)(m)^2^, Waist hip ratio = Waist (cm)/hip (cm), Increased waist circumference was defined by waist circumference > 90 cm (men) or > 80 cm (women), Hypertension at screening was defined as mean systolic blood pressure ≥ 140 mmHg, or diastolic blood pressure ≥ 90 mmHg on three measurements

* Median [25%, 75%]

**Table 2:** Prevalence of chronic kidney disease (CKD) by albumine creatinine ratio (ACR) and estimated glomerular filtration rate (eGFR)

Group	ACR ≥ 30 mg/g	EGFR < 60 ml/min/1,73	Total		Age ≥ 40 years old	
			N	Column %	N	Row %
No CKD	No	No	1777	87.2	827	46.5
CKD	Yes	No	212	10.4	136	64.2
	No	Yes	28	1.4	27	96.4
	Yes	Yes	20	1.0	20	100
Total			2037	100	1010	49.6

**Table 3:** Prevalence of chronic kidney disease by stage

		Urine albumin/creatinine ratio (mg/g)			Total	
Stage	EGFR (ml/min/1.73)	< 30	30–300	> 300	N	%
1	> 90	735	63	5	803	39.4
2	60–89	1042	125	19	1186	58.2
3a	45–59	27	11	3	41	2.0
3b	30–44	1	2	2	5	0.3
4	15–29	0	1	0	1	0.1
5	< 15	0	0	1	1	0.1
		1805	202	30	2037	100

### Prediction model for probability of CKD

We performed univariate analysis with the 23 nominal variables: (1) Age ≥ 50, (2) sex, (3) hypertension at screening, (4) increased waist circumference, (5) BMI > 23 kg/m^2^, (6) personal history (PH) of hypertension, (7) PH of diabetes, (8) PH of cardiovascular disease,(9) PH of congestive heart failure, (10) PH of stroke, (11) PH of kidney disease, (12) PH of urinary tract infection, (13) PH of kidney stone, (14) PH of nocturia, (15) PH of hematuria, (16) PH of alcohol drinking (either past or present), (17) PH of smoking (quit or present smoking), (18) PH of exercise, (19) PH of low salt intake, (20) family history (FH) of hypertension, (21) FH of diabetes, (22) FH of kidney disease, (23) FH of stroke.

After stepwise logistic regression, only 5 variables that showed statistical significance and had a high (> 1.7) odds ratio were retained for our final model: (1) age ≥ 50 (p < 0.0001), (2) hypertension on screening (p < 0.0001), (3) personal history (PH) of hypertension (p = 0.002), (4) PH of diabetes (p < 0.009) and (5) low salt diet (p = 0.002) (Table 4).

**Table 4:** Multivariate association of demographic and questionnaire variables with chronic kidney disease

	No CKD		CKD		Odds ratio (95% CI)
	(N)	(%)	(N)	(%)	
TOTAL	1774	100	260	100	
Age ≥ 50	491	27.7	152	58.5	2.0 (1.5–2.8)*
Male	816	46.0	113	43.5	0.9 (0.5–1.7)
Body mass index > 23 kg/m^2^	576	32.5	108	41.5	0.9 (0.7–1.3)
Increased waist circumference	251	14.1	72	27.7	1.4 (0.9–2.1)
Hypertension on screening	435	24.5	142	54.6	2.1 (1.5–2.9)*
History of diabetes	32	1.8	20	7.7	2.2 (1.1–4.2)*
History of hypertension	218	12.3	96	36.9	1.7 (1.2–2.5)*
History of kidney disease	192	10.8	33	12.7	0.9 (0.5–1.6)
History of hematuria	61	3.4	9	3.5	0.8 (0.3–1.6)
History of nocturia	449	25.3	103	39.6	1.4 (1.0–1.8)
History of cardiovascular disease	12	0.7	4	1.5	0.9 (0.2–3.0)
History of stroke	12	0.7	9	3.5	2.8 (1.0–7.3)
History of CHF	10	0.6	2	0.8	0.7 (0.1–3.2)
History of kidney stone	258	14.5	45	17.3	1.1 (0.6–1.7)
History of urinary tract infection	147	8.3	21	8.1	1.2 (0.7–1.7)
History of smoking	1199	67.6	181	69.6	0.7 (0.4–1.1)
History of drinking	700	39.5	100	38.5	1.3 (0.7–2.4)
History of low salt diet	63	3.6	33	12.7	2.3 (1.4–3.8)*
History of exercising	477	26.9	83	31.9	0. 8 (0.9–1.1)
Family history hypertension	783	44.1	105	40.4	1.5 (1.1–2.0)
Family history diabetes	184	10.4	28	10.8	0. 8 (0.5–1.3)
Family history of kidney disease	162	9.1	23	8.8	1.3 (0.8–2.1)
Family history stroke	221	12.5	45	17.3	1.7 (1.1–2.4)

Multivariate odds ratios for each of the variables is shown along with confidence intervals

* Indicates the variables which were used in the final model, based on a p < 0.001 and odds ratios > 1.7

The regression formula for probability of CKD was

$$\text{Probability}\,(\text{CKD})\, = \,1/(1 + \,\text{e}^{{ - \text{A}}} ),$$

where

$$\begin{aligned} \text{A} = \,[0.895]\; + \;(0.378)\; \times \;(age\; \ge \;50)\; + \;(0.372)\; \times \;(hypertension\,at\,screening)\; + \;(0.423)\; \times \;(PH\,of\,diabetes)\; \hfill \\ + \;(0.282)\; \times \;(PH\,of\,hypertension)\; + \,(0.397)\; \times \;(low\,\text{salt}\,\text{diet)} \hfill \\ \end{aligned}$$

Value in parenthesis = 1, if variable is true, and 0, if not.

To identify one person with CKD, the number of persons needed to screen (NNS) with a risk factor vs. without it was less by 67, 69, 70, 86, and 67% for measured hypertension, PH of diabetes, PH of hypertension, low salt diet, and age ≥ 50 years old, respectively (Table 5). Reference group was age younger than 50 and absence of each condition above for other factors (intercept 0.9).

**Table 5:** Final multivariate model for chronic kidney disease

Covariate	Number lab tested to find 1 CKD		Coefficient (β)	Standard error	Odds ratio (95%CI)	*p* value
	With covariate	Without covariate				
Hypertension at screening	4.1	12.4	0.37	0.08	2.1 (1.5–2. 9)	< 0.001
History of diabetes	2.1	8.3	0.42	0.16	2.3 (1.2–4.4)	0.01
History of hypertension	3.2	10.6	0.28	0.09	1.8 (1.2–2.5)	0.002
Low salt diet	2.9	20.9	0.40	0.13	2.2 (1.3–3.6)	0.002
Age ≥ 50	4.2	12.9	0.38	0.08	2.1 (1.6–2.9)	< 0.001

We chose probability > 0.389 in our regression model as a suggested threshold for confirmatory laboratory screening for CKD. With this threshold, the sensitivity for CKD was 13.8%, specificity 98.6%, positive predictive value 60%, and positive likelihood ratio 10.25. At this threshold, 2.9% of individuals were flagged for laboratory confirmatory screening.

We defined which discreet patterns of “yes” and “no” on the paper questionnaire yielded a probability of CKD of > 0.389 (Table 6). If there were four or more positive answers, the threshold of more than 0.389 was met and the subject was likely to have CKD by lab confirmatory testing. If there were three positive answers, and the subject age ≥ 50 years old, or the subject had hypertension despite a low salt diet, the threshold was also exceeded. The ability to use patterns of responses on a questionnaire to determine increased risk of CKD obviates the need for complex mathematical calculations in the field and lends itself to self-referral.

**Table 6:** The patterns of answering 5 questions and the predicted probability of more than 0.389

	Hypertension at screening	History of diabetes	History of hypertension	Low salt diet	Age ≥ 50	Number positive answers	Probability of ckd ≥ 0.389
1	*Yes*	*Yes*	*Yes*	*Yes*	*Yes*	5	*Yes*
2	No	*Yes*	*Yes*	*Yes*	*Yes*	4	*Yes*
3	*Yes*	No	Yes	Yes	Yes	4	*Yes*
4	*Yes*	*Yes*	*Yes*	No	*Yes*	4	*Yes*
5	No	No	*Yes*	*Yes*	*Yes*	3	*Yes*
6	No	*Yes*	*Yes*	No	*Yes*	3	*Yes*
7	Yes	No	No	*Yes*	*Yes*	3	*Yes*
8	*Yes*	*Yes*	No	No	*Yes*	3	*Yes*
9	*Yes*	No	*Yes*	No	*Yes*	3	*Yes*
10	*Yes*	No	*Yes*	*Yes*	No	3	*Yes*
11	Yes	Yes	Yes	No	No	3	No
12	No	Yes	Yes	Yes	No	3	No
13	No	No	No	Yes	Yes	2	No
14	No	No	Yes	No	Yes	2	No
15	No	No	Yes	Yes	No	2	No
16	No	Yes	No	No	Yes	2	No
17	No	Yes	No	Yes	No	2	No
18	No	Yes	Yes	No	No	2	No
19	Yes	No	Yes	No	No	2	No
20	Yes	No	No	Yes	No	2	No
21	Yes	No	No	No	Yes	2	No
22	Yes	No	No	No	No	1	No
23	No	Yes	No	No	No	1	No
24	No	No	Yes	No	No	1	No
25	No	No	No	Yes	No	1	No
26	No	No	No	No	Yes	1	No
27	No	No	No	No	No	0	No

The italics marked the patterns with probability of CKD > 0.389

### Receiver operating characteristic (ROC) curve

**Fig. 2:**
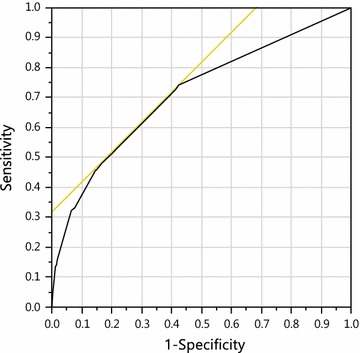
Receiver operator characteristic curve

The area under the receiver operator curve (AUC-ROC) was 0.71, using the reference group of individuals younger than 50 and absence of the risk factors above. This indicated that the discrimination of the model was fair (Fig. 2).

### The persistence of abnormal lab tests

We repeated the screening within 3 months on 189 out of 260 participants who had either ACR > 30 mg/g, or eGFR < 60 ml/min/1.73 m^2^. Repeated screens had the same proportion of microalbuminuria to macroalbuminuria as the original sample. 61% of the patients had persistent albuminuria and 67.6% of them had persistent eGFR < 60 ml/min/1.73 m^2^. 63.5% of individuals, who met criteria for CKD on initial screening, had CKD confirmed on second screening. Therefore, the estimated prevalence of CKD was 8.1% if based on persistent laboratory abnormalities rather than 12.76% based on a single screen.

### Cost effectiveness

The cost of lab tests (urine albumin/creatinine ratio, and serum creatinine) is 2.2 USD per person in Vietnam in 2015. Without a pretest questionnaire, the cost of testing 10,000 persons would be 22,000 USD or 27.16 USD for each of the 810 confirmed CKD cases. If the questionnaire should be used in 10,000 people, the total cost of further confirmatory laboratory tests on the 290 individuals expected to meet the threshold would be just 638 USD. The 5.8 USD per each of the 110 cases expected to be confirmed by this strategy compares to the 27.16 USD per confirmed case without using a questionnaire (Table 7).

**Table 7:** Estimated cost for lab tests for 10,000 participants with or without questionnaire

	N	CKD by lab	Total cost for lab tests (2.2 USD/person)	Cost per confirmed case
Population sample without questionnaire	10,000	810	$22,000	$27.16
Subset with risk threshold by questionnaire	290	110	$638	$5.80

## Discussion

The prevalence of chronic kidney disease in our sample was 12.76%, which was similar to other countries using the same definition (Urine ACR ≥ 30 mg/g or eGFR < 60 ml/min/1.73); 12.7% in England [19], 10.2% in Norway [20], 10.8% in China [21]. Different CKD prevalence has been found in surveys where different markers were used for eGFR evaluation (CKD-EPI equation 2009 instead of MDRD equation, or cystatin C for eGFR instead of serum creatinine) [19, 22]. The prevalence of CKD among the ≥ 40 years old participants in our study was 18.1%, which was higher than the China study with the same age group (11.3%) [23]. The percentage of hypertension (both by history and measured at screening time) in our sample was 32.11%, which was similar to other studies, 28.5 to 29% in the US [5, 24], 44.4% in Norway [20], 27.9% in China [21]. The prevalence of CKD among the subset of our patients who have the same risk factors found to predict CKD as in the KEEP study (history of hypertension, diabetes, family history of hypertension, diabetes and chronic kidney disease) was 163/1145 (14.2%). This was lower than the prevalence in the KEEP studies in the US (27.1%) [5] as well as in Japan [7] and Mexico [6]. However, 112/260 (43.1%) of the CKD patients didn’t have either hypertension on screening or history of hypertension, history of diabetes, or BMI more than 30 kg/m^2^, which was similar to the International Society of Nephrology Screening Programs in China, Mongolia, and Nepal [25]. Other factors might contribute to the CKD prevalence in our population, such as maternal malnutrition with ensuing low birth weight and low nephron endowment and increase in nephropathies related to infection, toxic agents [26, 27].

We have developed a method based on 5 characteristics (age ≥ 50, PH of hypertension, PH of diabetes, hypertension at screening and on low salt diet) to screen kidney disease in our population. Other characteristics expected to be correlated with CKD did not reach statistic significance but they depended on the prior health records and individual self reporting. Four of our 5 characteristics (age ≥ 50, hypertension, PH of hypertension, PH of diabetes) were known as risk factors for CKD [27], and contributed to the score of predicting CKD in other studies [13, 14].

In this study we found a paradoxical relationship between low salt diet and CKD. 96/2037 subjects (4.71%) in our study self reported intentional use of low salt diet. Low salt diet was found to be an independent risk factor for CKD in the multivariate analysis. We did not use the 24 h dietary history or timed urinary sodium measurement to estimate the amount of salt intake. No reason for or duration of low salt intake was determined. Whether low salt diet being part of any chronic disease therapy wasn’t known. Those subjects with low salt diet also had other lifestyle modifications (less alcoholic drink, less smoking, more doing exercise than normal salt intake group). A reduction in population salt intake will lower blood pressure and thereby reduce stroke and fatal coronary heart disease in adults. Other co-morbidity factors in those 96 subjects were statistically higher than the normal salt diet group (57.29% older than 50, 58.33% had hypertension on screening or history of hypertension, 45.83% history of nocturia, 20.83% history of CKD, 8.3% history of diabetes, 6.25% had history of CHF or CAD). Therefore, the association of low salt diet with CKD in our study is puzzling, but may be a covariate of a hidden factor not measured in this study.

### Limitations

The transportability of our score remains to be validated in other rural populations coincidentally with using lab tests for CKD diagnosis and adding low salt diet measurement. We cannot rule out the possibility that other co-morbid conditions might also have been misclassified or under-ascertained. The study used only a single random ACR and single eGFR for CKD diagnosis (12.76%) which overestimated the prevalence of CKD required the persistent abnormality either of them (8.1%). In NHANES III study, the reproducibility of data on microalbuminuria on the second visit within 2 months of the initial examination was only 57%, while the repeat serum creatinine showed good agreement with the initial measurement [28]. The cost of lab tests in Vietnam (including urine albumin creatinine ratio, and serum creatinine) was 2.2 USD in 2015. With limited available health resources (6.0% of GDP in 2013) [15], out-of-pocket payments remain high, and any savings are worthy of consideration. By using the questionnaire before laboratory testing, we could affect a 78% decrease of the cost per each confirmed case of CKD. The strategy of using a simple questionnaire with five variables which could be repeated multiple times without adding any cost is helpful as a screening aid for CKD in the rural areas of Vietnam.

Although the sensitivity of this questionnaire is low (13.8%), the positive predictive value is high enough (60%) to detect a significant number of people at risk. There are fewer than 1.22 physicians and 2.3 total health care workers (including nurses and midwives) per 1000 population in Viet Nam [15]. Travel from rural areas to central laboratories is expensive and time consuming. This questionnaire could guide local health workers and patients about individuals at higher risk of CKD.

## Conclusions

In conclusion, this questionnaire, along with routine clinical evaluation, is a simple, cost-effective tool to assess CKD which can be implemented in rural areas of Vietnam. It also gives more effectiveness and productivity to each penny spent for health care system in Vietnam.

## Abbreviations

CKD: chronic kidney disease
ACR: albumin-creatinine ratio
eGFRMDRD: estimated GFR by 4 variable MDRD equation
BMI: body mass index
CAD: coronary artery disease
CHF: congestive heart failure
PH: personal history
FH: family history
CIs: confidence intervals
